# Molecular residual disease-based novel modality of postoperative management for non-small-cell lung cancer (REMODEL): Protocol for a prospective multicenter study

**DOI:** 10.1515/jtim-2026-0023

**Published:** 2026-05-16

**Authors:** Xiaoqiu Yuan, Ruoyi Jin, Yunchu Wei, Yue He, Lin Weng, Zifan Li, Jun Aiden Wang, Xinrui Li, Junnan Xu, Jian Bai, Yun Wang, Qingna Zhang, Na Zhou, Yanyan Hou, Guangxi Wang, Jiatao Zhang, Rong Yin, Ziming Li, Yun Li, Fan Yang, Jun Wang, Kezhong Chen, On behalf of the REMODEL Study

**Affiliations:** Research Unit of Intelligence Diagnosis and Treatment in Early Non-small Cell Lung Cancer, Chinese Academy of Medical Sciences, 2021 RU002, Peking University People's Hospital, Beijing, China; Thoracic Oncology Institute, Peking University People's Hospital, Beijing, China; Department of Thoracic Surgery, Peking University People's Hospital, Beijing, China; Peking University Health Science Center, Beijing, China; Kanghui Biotech Co., Ltd., Liaoning Province, China; Department of Pathology, School of Basic Medical Sciences, Institute of Systems Biomedicine, Peking-Tsinghua Center for Life Sciences, Peking University Health Science Center, Beijing, China; Guangdong Lung Cancer Institute, Guangdong Provincial People's Hospital, Guangdong Academy of Medical Sciences, Guangzhou, Guangdong Province, China; Department of Thoracic Surgery, Jiangsu Cancer Hospital, Jiangsu Institute of Cancer Research, the Affiliated Cancer Hospital of Nanjing Medical University, Jiangsu Key Laboratory of Molecular and Translational Cancer Research, Collaborative Innovation Center for Cancer Personalized Medicine, Nanjing, Jiangsu Province, China; Department of Shanghai Lung Cancer Center, Shanghai Chest Hospital, Shanghai Jiao Tong University School of Medicine, Shanghai, China; Clinical Medical College, Hebei University, Baoding, Hebei Provine, China

**Keywords:** non-small-cell lung cancer, minimal residual disease, recurrence monitoring, adaptive postoperative management

## Abstract

**Background and Objectives:**

Circulating tumor DNA (ctDNA)-based molecular residual disease (MRD) has been confirmed to predict postoperative recurrence of early-stage non-small-cell lung cancer (NSCLC), outperforming conventional imaging modalities by improving diagnostic efficiency and eliminating radiation-related risks. Integrating ctDNA with other liquid biopsy omics methods might further increase monitoring accuracy. In addition, patients with longitudinal undetectable MRD exhibit a favorable outcome and may be overtreated after radical surgery, while those traditionally not recommended for adjuvant treatment but with positive MRD may have not received appropriate disease management. This prospective real-world trial involving ten clinical centers across China aims to develop a multiomics noninvasive platform for recurrence prediction and to determine whether postoperative management guided by ctDNA is noninferior to standard care.

**Methods:**

We will recruit pathologically confirmed TNM stage IA-IIIA (8^th^ edition) NSCLC patients who are undergoing radical resection. Eligible participants will be divided into a standard treatment group and a ctDNA-guided group depending on their preference. Patients treated with or without neoadjuvant therapy will be studied in parallel. In the ctDNA-guided group, adjuvant therapy can be avoided for patients whose MRD is undetected at 1 month after surgery, and an additional requirement is to achieve a major pathological response for patients receiving neoadjuvant therapy. Imaging and MRD monitoring will be performed every 3–6 months for 3 years of follow-up, and postoperative management will be determined by the MRD results at each time point. Adjuvant therapy will be stopped if the patient's MRD status is continuously negative; otherwise, a treatment plan will be initiated after discussion among a multidisciplinary team (MDT). The recruitment phase began in April 2025, and 916 patients will be enrolled.

**Discussion:**

This prospective, multicenter, real-world study provides innovative techniques and is the first to confirm the instructive value of MRD for postoperative management of resectable NSCLC.

## Introduction

Lung cancer is among the malignancies with the highest mortality worldwide.^[1, 2]^ According to the 2011–2019 database from the International Association for the Study of Lung Cancer (IASLC), early-stage cases account for approximately 60% of non-small cell lung cancer (NSCLC).^[3]^ While curative surgery is the first-line treatment for these patients, more than 30% experience postoperative recurrence.^[4, 5]^ However, conventional imaging-based recurrence monitoring has reached a sensitivity bottleneck, with a reported false-positive rate of 17%–22%.^[6, 7]^ Multicenter clinical trials have shown that compared with X-ray, computed tomography (CT)-based surveillance does not improve patient outcomes. Recent studies further indicate that CT scans significantly increase the risk of brain and hematologic malignancies.^[8, 9]^ There is an urgent need for a more sensitive, noninvasive, and radiation-free approach to monitor postoperative progression.

Liquid biopsy-based minimal residual disease (MRD) detection is emerging as a precise tool for the postoperative management of solid tumors,^[10, 11]^ among which circulating tumor DNA (ctDNA) demonstrates promising roles in early-stage NSCLC.^[12]^ A positive state of ctDNA at a landmark (3 days–1 month after surgery) has shown hazard ratios of 2.9–43.4 for disease-free survival (DFS),^[13]^ and longitudinal monitoring achieves a higher sensitivity of 99.9% for relapse detection, with a median lead-time of 180–299 days compared with chest CT.^[14]^ However, the accuracy of ctDNA detection clearly decreases in patients with a low tumor burden or in nonshedders,^[14, 15]^ and the time point of blood sample collection during long-term surveillance is also not fully standardized. With the ongoing applications of other liquid biopsy omics methods in MRD detection, including plasma metabolomics,^[16]^ methylation,^[17]^ and fragmentomics,^[18]^ integrated approaches hold promise for further increasing the sensitivity of recurrence surveillance. Thus, the first step of this study aims to leverage multiomics technologies to establish a highly accurate and accessible MRD detection system for NSCLC.

As for adjuvant therapy, the overall aim is to improve patient survival without unacceptable diminishment of quality of life (QoL).^[19]^ However, TNM staging-based strategies are relatively homogeneous and fail to fit all patients. For NSCLC patients with EGFR-sensitive mutations in stages IB-IIIA, the National Comprehensive Cancer Network (NCCN) guidelines recommend 3-year adjuvant TKI therapy.^[20]^ Nonetheless, the ADAURA study revealed that 33% of patients in the control group remained disease free for 5 years,^[21]^ suggesting overtreatment in some patients. Additionally, increased relapses post-TKI discontinuation also imply a suboptimal treatment duration.^[22]^ A similar issue applies to driver-negative cases. In addition, the indication for adjuvant therapy in patients who achieve pathological complete response (pCR) following neoadjuvant treatment remains controversial.^[23]^ More precise adjuvant treatment strategies are needed.

Given the monitoring efficacy of ctDNA, investigators have explored novel modalities of ctDNA-guided intensification/de-escalation for standard treatment (adaptive adjuvant therapy). A randomized controlled trial for colorectal cancer has proven the feasibility of ctDNA-guided reduction in adjuvant chemotherapy without compromising survival.^[24]^ In advanced NSCLC, an adaptive de-escalation TKI strategy for patients with no lesions after local consolidation therapy and a negative ctDNA test result is feasible.^[25]^ Although exploratory analyses in early-stage NSCLC suggest that only patients with positive ctDNA results may benefit from adjuvant therapy,^[26, 27]^ whether a ctDNA-guided adaptive strategy is suitable for routine clinical practice in the postoperative setting remains unclear. Therefore, this MRD-based modality will be evaluated in the second step of this study. Through this multicenter real-world observational-interventional study design, we aim to establish the first noninvasive and systemic strategies for the precise postoperative management of NSCLC.

## Overview of the study design

This study includes two interlinked parts: an observational substudy and a prospective interventional substudy. Patients will be recruited from 10 tertiary clinical centers in China (Table 1), including stage IA-IIIA NSCLC patients who are initially treated or receiving neoadjuvant therapy. Each site has a high volume of eligible patients (> 1000 patients with early-stage NSCLC each year) and has expertise in enrolling patients and the ability to collect and store biospecimens. Patients will be followed after surgery, and blood collection will be performed at prespecified time points.

**Table 1:** Medical centers of the REMODEL study

Peking University People’s Hospital
Guangdong Provincial People’s Hospital
Jiangsu Cancer Hospital
Shanghai Chest Hospital
The Second Xiangya Hospital, Central South University
Shenzhen Third People’s Hospital
Zhongshan Hospital, Fudan University
Peking University First Hospital
Wuhan Union Hospital
Shandong Cancer Hospital

The observational substudy aims to develop a novel multidimensional approach for noninvasive postoperative recurrence monitoring of NSCLC by innovatively integrating multiomics data, including clinicopathological features, plasma metabolomics, proteomics, cell-free DNA (cfDNA) features and radiomics, through artificial intelligence algorithms. Model development will leverage perioperative blood samples from early-stage NSCLC patients in a retrospective cohort, with external validation performed using samples of prospectively recruited patients.

The prospective interventional substudy will evaluate the feasibility, safety and clinical efficacy of ctDNA-MRD-based treatment intensification/de-escalation strategies in real-world settings while exploring the association between postoperative ctDNA-MRD monitoring dynamics and clinical outcomes. We will divide patients into standard treatment and ctDNA-MRD-guided groups, primarily comparing the two-year DFS rate and the total cost of treatment 24 months post-operative, as well as externally validating the sensitivity of the novel monitoring method established by the observational substudy.

## Objectives

### The primary aims of this study are as follows:

(1) To develop a novel multidimensional approach with increased sensitivity for noninvasive postoperative recurrence monitoring of NSCLC;

(2) To establish a ctDNA-MRD-guided postoperative management modality for NSCLC patients;

(3) To establish a national database and a sustainable digital platform for postoperative tracking of NSCLC.

Secondary aims of interest include the following:

(1) To explore the association between ctDNA changes during adjuvant therapy and patient outcomes;

(2) To investigate the association between ctDNA features and patient outcomes in the neoadjuvant setting.

## Observational substudy: A novel multiomics noninvasive platform to monitor postoperative recurrence

### Settings

**Figure 1:**
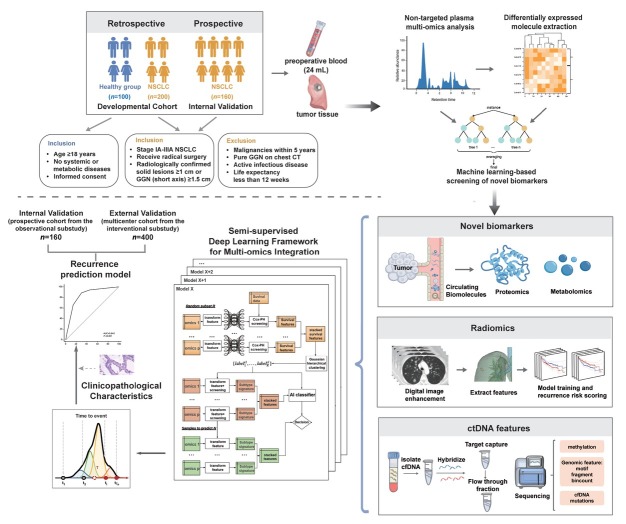
Design of the observational substudy. The retrospective cohort comprises healthy controls (*n* = 100) and NSCLC patients (*n* = 200). This cohort will be used to establish a noninvasive platform for postoperative recurrence monitoring, and AI-based integration of ctDNA, radiomic features, and novel biomarkers identified through metabolomics and proteomics analysis will be leveraged. Internal validation will be performed using the prospective cohort from the observational substudy (*n* = 160), with external validation conducted through the multicenter interventional substudy (*n* = 400; see the intervention substudy section for details).

This observational substudy comprises retrospective and prospective components (Figure 1). The retrospective cohort of 200 treatment-naive NSCLC patients and 100 matched cancer-free adults was derived from the previously established Pulmonary Nodule Plasma Proteomic and Metabolomic Database of Peking University People’s Hospital (Ethics Approval No. 2021PHB150-001; ClinicalTrials.gov Identifier: NCT04948047), while the prospective cohort of 160 NSCLC patients will be consecutively enrolled from the same institution. First, we will screen novel MRD biomarkers in the retrospective cohort and perform exploratory recurrence detection modelling that integrates clinicopathological features, plasma metabolomics, proteomics, cfDNA signatures and radiomics. We will subsequently internally validate the new model on prospective components and further conduct external validation using samples from the interventional substudy.

### Patient enrollment

The inclusion and exclusion criteria are listed in Table 2. Patients will be identified and enrolled by clinics at the enrollment sites. During the clinic visit or hospitalization, the clinical researcher will confirm eligibility, ensure that the patient provides informed consent, and collect relevant study data and blood samples.

**Table 2:** Inclusion and exclusion criteria for the observational substudy

**Retrospective enrollment of cancer-free individuals (*n* = 100)**	
Inclusion	
	(a) Age ≥18 years;
	(b) No systemic or metabolic diseases;
	(c) Signed informed consent.
**Retrospective enrollment of patients with NSCLC (*n* = 200)**	
Inclusion	
	(a) Adults (≥18 years) with pathologically confirmed TNM stage IA-IIIA (8th edition) NSCLC that are undergoing radical surgery;
	(b) Availability of matched tumor tissue, blood samples and accessible imaging records;
	(c) No history of other malignancies within the past 5 years;
	(d) No neoadjuvant therapy;
	(e) Signed informed consent.
**Prospective enrollment of patients with NSCLC (*n* = 160)**	
Inclusion	
	(a) Adults (≥18 years) with clinical TNM stage IA-IIIA (the 8th edition) NSCLC that are scheduled for radical surgery;
	(b) Radiologically confirmed solid lesions with diameters ≥1 cm * or ground-glass nodules (GGN) with short-axis diameters ≥1.5 cm;
	(c) Signed informed consent.
Exclusion	
	(a) Pure GGN observed via chest CT;
	(b) History of any other malignancy within 5 years;
	(c) Has active infectious diseases;
	(d) Failure to undergo radical surgery;
	(e) Pathologically confirmed non-NSCLC;
	(f) Pathologically confirmed TNM stage IIIB-IV (8th edition);
	(g) Life expectancy less than 12 weeks.

^*^Solid lesions with long-axis diameters measuring 1–1.5 cm also require short-axis diameters ≥1 cm to preserve adequate samples for baseline detection. NSCLC: non-small cell lung cancer; GGN: ground-glass nodule; CT: computed tomography.

### Data and sample collection

#### Data collection

Demographic characteristics, clinical profiles, and preoperative images will be obtained through electronic medical record review. Comprehensive documentation includes treatment details (surgical procedures and adjuvant therapy), therapeutic efficacy, and treatment-related adverse events. Pathological data from surgical specimens, along with postoperative imaging examinations and prognostic outcomes during follow-up, will be prospectively recorded in a centralized research database.

#### Collection and storage of biospecimens

Twenty-four milliliters of preoperative blood samples and matched tissue specimens (for customized MRD sequencing panel development) will be collected. Venous blood will be allocated as follows: 4 mL will be transferred to EDTA-coated tubes for plasma metabolomic and proteomic profiling (processed within 2 h with protease inhibitor cocktail addition after centrifugation), and 20 mL will be transferred to Streck cell-free DNA BCT tubes for ctDNA analysis (centrifuged within 72 h). Equal-volume samples will be taken from one side of the tumor using a skin perforator. For smaller tumors, researchers will consider sampling from various locations on the opposite side. Samples from at least two tumor regions will be separated by 0.3 cm to 1.0 cm, depending on the tumor size. Border samples will be collected at the tumor margin to ensure that both tumor and normal samples are obtained. Paired normal tissue will be taken at least 2 cm from the tumor edge. Finally, the central coordinates of each sampling point will be recorded using a double-scale square and documented with photographs. All aliquots will be cryopreserved at −80 °C until batch analysis.

### Data analysis

#### Multiomics profiling

Multiomics profiling, including radiomics-based recurrence prediction, nontargeted plasma metabolomics and proteomics, cfDNA methylation and genomic feature analysis, and individualized ctDNA detection, will be conducted. The detailed methods are provided in Supplementary material 1.

#### Optimization of plasma proteomic and metabolomic features characterizing lung cancer lesions

In the retrospective cohort, we will perform untargeted proteomic and metabolomic profiling on plasma samples from lung cancer patients and healthy controls to identify lung cancer-associated signatures as potential MRD biomarkers. Initial selection will be performed using the Wilcoxon rank-sum test with FDR correction (adjusted *P* < 0.05). Candidate selection criteria include mass spectrometry-detectable features with stable retention time, reproducible peak intensity, interpretable fragmentation patterns, and clinically relevant half-life, optimized through machine learning algorithms.

For model-driven feature optimization, the differential molecules will be first evaluated for their contribution to classification using a support vector machine (SVM) model, and the top 100 features will be sequentially added according to their importance scores to construct stepwise classification models. At each step, model performance will be evaluated using fourfold cross-validation, which will be repeated 500 times to ensure stability and robustness. The average classification accuracy across all iterations will be used to determine the optimal number of features for each omics layer. Based on the point of peak model performance and cross-validation consistency, a final subset of 30 to 50 informative features will be selected and used for downstream integration. This two-stage process is designed to balance dimensionality reduction with predictive efficiency and biological interpretability.

#### Development and validation of a multidimensional model for noninvasive recurrence prediction

This study will employ a semisupervised DeepProg framework^[28]^ to synthesize plasma metabolomic/proteomic profiles, radiomic recurrence scores, and multianalyte cfDNA features in the retrospective patient cohort. The semisupervised DeepProg framework combines autoencoder-based dimensionality reduction and Cox proportional hazards modeling. For each omics layer, the top-ranked features will be preselected on the basis of univariate survival associations and effect sizes. These features will be embedded into latent representations using separate autoencoders and then fused into a unified risk prediction model. The DeepProg architecture allows the integration of incomplete data across layers and enables survival risk stratification based on molecular profiles. The final prognostic model will combine multidimensional risk scores with TNM staging through Cox proportional hazards regression. A threshold for recurrence risk stratification will be optimized in the training cohort to categorize patients into high- and low-risk groups, and its prognostic utility will be validated in the test and validation cohorts *via* Kaplan^‒^Meier analysis. We will also assess the sensitivity, specificity, accuracy and C-index. The model will be externally and respectively validated on a prospective patient cohort and a multicenter cohort from subsequent phases of this study to confirm its reproducibility and generalizability.

#### Sample size calculation

Sample size determination was guided by a literature review and practical considerations, with 200 lung cancer patients and 100 healthy controls enrolled retrospectively for prediction model development. The sample size of the independent prospective cohort should consider recurrence prediction validation and 2-year DFS comparisons. On the basis of the calculations in Supplementary material 2, we plan to prospectively enroll 160 patients. Enrollment continuation will be determined by the investigator after the predefined sample size is met.

## Interventional substudy: MRD-based adaptive postoperative management for lung cancer

### Settings

**Figure 2:**
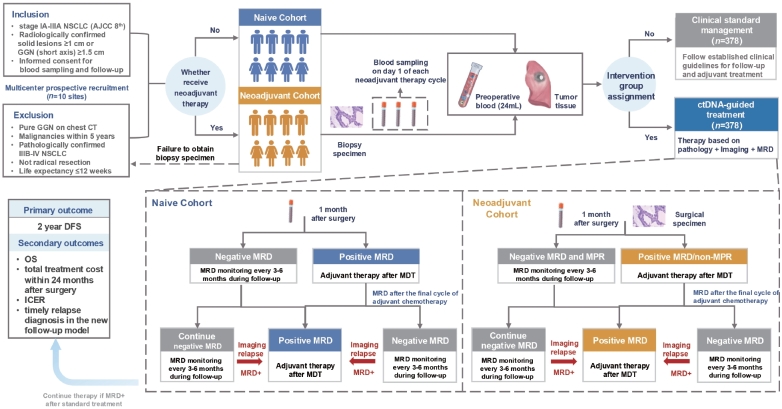
Design of the interventional substudy. Participants will be stratified by preoperative treatment status (neoadjuvant therapy or upfront surgery). Allocation will follow real-world preferences to either the clinical standard management group (*n* = 378) or the ctDNA-MRD-guided treatment group (*n* = 378), with longitudinal monitoring at 3–6-month intervals. In the MRD-guided arm, postoperative management will be tailored according to serial MRD dynamics and integrated with pathological response assessments in neoadjuvant therapy subgroups. Two-year DFS will be evaluated as the primary outcome.

This prospective multicenter real-world study aims to evaluate whether compared with standard management, ctDNA-MRD-guided postoperative surveillance and adjuvant therapy strategies improve clinical outcomes and cost-utility in patients with stage IA-IIIA NSCLC. We will prospectively enroll 756 patients across 10 clinical centers. Participants will undergo real-world preference allocation to either the standard treatment group or the ctDNA-MRD-guided treatment group, with longitudinal monitoring at 3–6-month intervals. The primary endpoint is the two-year DFS rate (Figure 2).

### Patient enrollment

Patients who have not been treated preoperatively or who have received neoadjuvant therapy will be recruited separately, with detailed inclusion/exclusion criteria provided in Table 3. During clinic visits or hospitalizations, researchers will confirm the eligibility of patients, obtain informed consent, and collect relevant study data and blood samples.

**Table 3:** Inclusion and exclusion criteria for the interventional substudy

**Patients without neoadjuvant therapy (*n* = 609)**	
Inclusion	
	(a) Adults (≥18 years) with clinical stage IA-IIIA (8th edition) NSCLC that are scheduled for curative surgery;
	(b) Radiologically confirmed solid lesions with diameters ≥1 cm* or GGN with short-axis diameters ≥1.5 cm;
	(c) Signed informed consent.
Exclusion	
	(a) Pure GGN observed via chest CT;
	(b) History of any other malignancy within 5 years;
	(c) Has active infectious diseases;
	(d) Failure to undergo radical surgery;
	(e) Pathologically confirmed non-NSCLC;
	(f) Pathologically confirmed TNM stage IIIB-IV (8th edition);
	(g) Has received neoadjuvant therapy;
	(h) Life expectancy less than 12 weeks.
**Patients receiving neoadjuvant therapy (*n* = 147)**	
Inclusion	
	(a) Adults (≥18 years) with clinical TNM stage (8th edition) I-III NSCLC that are scheduled for neoadjuvant therapy;
	(b) Signed informed consent.
Exclusion	
	(a) History of any other malignancy within 5 years;
	(b) Has active infectious diseases;
	(c) Pathologically confirmed non-NSCLC;
	(d) Pathologically confirmed TNM stage IIIB-IV (8th edition) after neoadjuvant therapy;
	(e) Failure to obtain biopsy specimens before neoadjuvant therapy;
	(f) Failure to undergo radical surgery;
	(g) Life expectancy less than 12 weeks.

^*^Solid lesions with long-axis diameters measuring 1–1.5 cm also require short-axis diameters ≥1 cm to preserve adequate samples for baseline detection. NSCLC: non-small cell lung cancer; GGN: ground-glass nodule; CT: computed tomography.

### Data collection and testing

#### Data collection

Demographic characteristics, clinical profiles, and preoperative imaging findings will be obtained through electronic medical records review. Comprehensive documentation includes treatment details (surgical procedures, neoadjuvant therapy, and adjuvant therapy), therapeutic efficacy, and treatment-related adverse events. Pathological data from surgical specimens or biopsy samples, along with serial postoperative imaging examinations, ctDNA-MRD detection and prognostic outcomes during follow-up, will be prospectively recorded in a centralized research database.

#### Collection and storage of biospecimens

The participants will have 24 mL of blood collected 0–3 days before the operation, 3–10 days after surgery, 1 month after surgery, and every 3–6 months during the three years of follow-up. We will allow a maximum deviation of ±1 month around each scheduled postoperative draw to accommodate clinical logistics. Sensitivity analyses will be performed by excluding samples collected outside the ±2-week window and re-estimating MRD dynamics and their correlation with outcomes to confirm the robustness of temporal patterns. Additional peripheral blood samples will be collected within 1 week before neoadjuvant therapy initiation and on Day 1 of each treatment cycle for patients receiving neoadjuvant regimens. For patients who require adjuvant therapy based on clinicopathological characteristics and MRD assessment, blood sampling will be conducted at the beginning of adjuvant therapy and immediately after the completion of adjuvant chemotherapy cycles, with subsequent sampling performed every 3–6 months. For patients deemed not to receive adjuvant therapy, regular blood monitoring will be performed at 3–6-month intervals.

Surgical specimens will be collected for tumor-informed ctDNA-MRD testing. The processing and preservation of the biospecimens are outlined in the observational substudy section.

#### MRD assay

In this study, we will use tumor-informed ctDNA detection, which is consistent with the methodology described in the *circulating tumor DNA assay* section.

### Interventions

#### Experimental arm (ctDNA-guided management)

In the ctDNA-guided arm, for treatment-naive patients, the results of ctDNA-MRD detected from blood before surgery, on Day 3 and 1 month after surgery will be provided to clinicians and patients 6–8 weeks after surgery. Notably, blood samples obtained on postoperative Day 3 may not be obtainable consistently in clinical practice. Thus, patients whose MRD is undetected 1 month after surgery will enter surveillance with blood sampling every 3–6 months during follow-up without additional adjuvant therapy. Blood samples obtained 3 days after surgery may serve as an alternative source of data in circumstances where blood sampling 1 month after surgery is not performed as planned. Furthermore, the concurrent availability of specimens from day 3 and 1 month after surgery facilitates a more robust assessment, wherein the Day 3 data provide supplementary context. Adjuvant therapy will be initiated if the patient’s MRD status is positive or if progression is suggested by imaging. The MDT will recommend specific treatment strategies on the basis of prior therapy, histology, and gene mutation. Treatment decisions will be individualized but guided by national guidelines to ensure consistency across centers.

For neoadjuvant-treated patients, ctDNA-MRD results of blood samples collected before/during neoadjuvant therapy, before surgery, and after surgery will be analyzed within 4–6 weeks after surgery. Patients will receive adjuvant therapy if any of the following criteria are met: the major pathological response is not reached (non-MPR) or postoperative ctDNA positivity conditions are met. Only patients who achieve MPR and negative ctDNA results 1 month after surgery will avoid adjuvant therapy, with surveillance of ctDNA-MRD detection performed every 3–6 months. Therapy will begin if ctDNA becomes positive.

If adjuvant therapy is indicated postoperatively after assessment, the MRD assessment will be performed after chemotherapy is completed (within weeks 3–4 in the final cycle of treatment).^[29]^ If MRD is positive, the patient will continue adjuvant therapy; otherwise, maintenance treatment will be stopped. Subsequent MRD monitoring should be performed every 3–6 months, with positive results or imaging progression triggering MDT discussion for treatment and negative results confirming therapy termination. If MRD persists after standard adjuvant cycles, the treatment duration will be extended.

#### Control arm (standard treatment)

In the control arm, patients will receive standard postoperative management and follow-up. For patients receiving regular MRD testing, their clinicians will be blinded to the initial ctDNA results, which can be disclosed upon request after 6 months.

#### Discontinuing or modifying the intervention

Below are predefined cases in which patients will be removed from the study intervention but will continue routine clinical care, such as clinic visits, labs, and imaging, per their clinicians:

(1) The patient cannot comply with protocol requirements.

(2) The patient develops a malignancy that requires treatment, which will interfere with this study.

(3) The patient is lost in follow-up.

### Outcomes

The primary endpoint of this clinical trial is the two-year DFS rate after surgery, defined as the interval from the surgical date to objectively confirmed disease recurrence or death from any cause.

Secondary outcomes of interest include (1) overall survival (OS), (2) total treatment cost 24 months after surgery, (3) incremental cost effectiveness ratio (ICER), (4) sensitivity of the novel recurrence prediction model developed in the observational substudy, (5) timely diagnosis of recurrence in the new follow-up model developed by the observational substudy, and (6) ctDNA characterization during neoadjuvant/adjuvant therapy and its associations with clinical outcomes, including short-term efficacy (radiological and pathological response) and long-term survival. Total treatment cost refers to the expense of lung cancer-related treatment for the patient during the research period, including the drug cost, follow-up treatment cost, drug management cost (consultation fee, antitumor drug allocation fee, intravenous infusion fee, nursing fee, *etc*.), cost of follow-up laboratory tests and imaging, and management cost of adverse reactions of grade 3 or greater, with an incidence rate of more than 5%.

### Statistical analysis

Continuous variables will be described as the mean (with standard deviation [SD]) or median (with interquartile range [IQR]) depending on whether they fit a normal distribution. Categorical variables will be described as counts and percentages. Comparisons between groups will be performed using appropriate statistical tests (*chi-square* test for categorical variables; Student’s *t* test or Mann^‒^ Whitney *U* test for continuous variables). To address the potential confounding variable introduced by preference-based group allocation, we will implement propensity score methods, including propensity score matching (PSM) and inverse probability of treatment weighting (IPTW).^[30]^ Multivariate analyses for covariate correction will be conducted using logistic regression, linear regression, and Cox proportional hazards regression, depending on the type of outcome variables. Variance inflation factors (VIFs) will be assessed *via* correlation matrices, with variables with a VIF < 3.0 retained. Firth’s penalized maximum likelihood bias reduction method for Cox regression will be implemented to handle the monotone likelihood. All analyses will be performed using R (version later than 4.4.0), and a two-tailed p value of < 0.05 will be considered to indicate statistical significance. Detailed methodologies for outcome analysis, subgroup evaluation, and sample size estimation are provided in Supplementary materials 3–4.

## Discussion

Currently, the use of MRD for monitoring postoperative recurrence of lung cancer requires a high level of accuracy, and high-quality evidence supporting the predictive value of MRD in adjuvant therapy is lacking. Postoperative chemotherapy, long-term targeted therapy, and immune checkpoint inhibitor treatments impose certain toxic side effects and financial burdens on patients. Therefore, first, this study primarily aims to develop a recurrence monitoring method that integrates multiomics data to improve sensitivity. Additionally, this is the first prospective, interventional, multicenter real-world study to explore the rationale of MRD-guided postoperative management for patients with resectable NSCLC.

Although this study adopts a preference-based allocation design, all participants will be enrolled under strict inclusion and exclusion criteria, ensuring a clinically homogeneous study population. This approach reflects real-world clinical practice where patient preference influences treatment choice. To minimize potential selection bias, we will collect detailed baseline variables and apply propensity score methods to adjust for measurable confounders. Additionally, we will perform multivariate analyses for covariate correction. Subgroup analysis will also control for baseline imbalances. Although this design may not support strict causal inference, it allows for a more pragmatic evaluation of MRD-guided management strategies in real-world clinical practice.

Studies have shown that MRD can be detected by liquid biopsies well before recurrence can be detected by standard clinical methods.^[31,32]^ Moreover, both plasma metabolomics^[33]^ and proteomics demonstrate strong correlations with cancer risk. With respect to radiomics, studies have reported that integrating radiological features with ctDNA detectability improves the prediction of NSCLC outcomes.^[34, 35]^ Multiomics integration has been used in early tumor detection and has shown high accuracy.^[36, 37]^ Therefore, our study pioneers the integration of plasma multiomics, radiomics, and clinicopathological features, which is crucial for increasing the sensitivity and accuracy of noninvasive monitoring methods for postoperative lung cancer recurrence.

Previous studies have revealed the immense potential of MRD in guiding postoperative adjuvant therapy. In the FLAURA2 study,^[38]^ for patients who were ctDNA positive at baseline, the addition of chemotherapy to standard osimertinib treatment further prolonged progression-free survival (PFS). However, for ctDNA-negative patients at baseline, combining chemotherapy did not provide an additional improvement in PFS. Another study^[39]^ revealed that compared with patients who were ctDNA-positive at either time point, NSCLC patients who were ctDNA-negative at both the neoadjuvant and postsurgical time points had significantly higher 18-month event-free survival rates, suggesting that combined MRD testing could help identify patients who may have been cured and thus who do not require additional adjuvant therapy. Nevertheless, these findings remain confined to exploratory analyses derived from observational studies, serving solely to hypothesize the therapeutic guidance value of ctDNA-MRD. In contrast, this study will systematically investigate the clinical feasibility, safety, and efficacy of MRD-guided adaptive strategies through comparative analysis with standard postoperative management. With respect to the reason for choosing 3–6 months as the MRD monitoring time, we considered clinical safety and feasibility. The European Society of Medical Oncology (ESMO) recommends the use of ctDNA assays for tumor patients ^[40]^ and reported that tumor-informed ctDNA detection generally progresses from ctDNA detection to clinical relapse in < 6 months. A TRACERx study ^[15]^ also verified that ctDNA surveillance every 3–6 months identified impending disease relapse in an additional 20% of patients whose ctDNA had been negative within 120 days after surgery. These findings demonstrated the value of this detection interval for early warning of recurrence. Moreover, longitudinal detection in prospective studies or randomized controlled trials of ctDNA-based MRD detection in resectable tumors occurred mostly 3–6 months after surgery, which may also be consistent with the follow-up time.^[41]^ However, whether this timepoint is the most appropriate needs further validation. The evaluation endpoints are formulated in accordance with the ESMO to define whether an intervention is clinically meaningful as a > 5% improvement in survival or an improvement in DFS with an HR < 0.65 (lower limit of the 95% confidence interval) for studies without mature survival data. Noninferior OS or DFS with reduced treatment toxicity or improved QoL can also be clinically meaningful.^[42]^

This study will help clarify the therapeutic value of MRD-guided postoperative management in lung cancer patients. If MRD-guided strategies result in superior patient outcomes, they could significantly alter clinical decision-making. If no significant difference in patient prognosis is observed but a notable reduction in adverse effects or treatment costs is achieved, incorporating MRD into clinical practice could still serve as a viable alternative, effectively ensuring timely intervention for high-risk subgroups while sparing patients from unnecessary therapy toxicity and thereby harmonizing survival benefits with quality-of-life preservation. From a broader perspective, this study exemplifies the convergence of translational medicine and artificial intelligence, providing a blueprint for real-world validation of liquid biopsy technologies. Elucidating the interplay between residual tumor biology and host responses advances our mechanistic understanding of cancer recurrence. The establishment of a nationwide NSCLC postoperative database and digital platform further facilitates future biomarker discovery and precision oncology research.

In summary, this study represents a transformative endeavour for redefining the postoperative management of early-stage NSCLC by bridging cutting-edge multiomics technologies with real-world clinical practice. The integration of plasma metabolomics, proteomics, radiomics, and ctDNA-based MRD monitoring will establish a novel paradigm for noninvasive recurrence surveillance and address the sensitivity limitations of current imaging-centric approaches. On the other hand, by pioneering a ctDNA-guided adaptive therapeutic strategy, this study will challenge the conventional “one-size-fits-all” adjuvant therapy modality and offer a customized framework to tailor interventions based on MRD dynamics, further contributing to the shift in global oncology practice from population-based guidelines to individualized biomarker-driven care.

## Supplementary Material

Supplementary Material Details
