# Supplementary material for: Molecular residual disease-based novel modality of postoperative management for non-small-cell lung cancer (REMODEL): Protocol for a prospective multicenter study
Source: J Transl Int Med. 2026 May 16;14(3):470–80. doi: 10.1515/jtim-2026-0023 (PMC13320532; doi:10.1515/jtim-2026-0023)
Supplement: Supplementary file 1 — Supplementary Material Details [file jtim-2026-0023_sm.pdf]

Supplementary materials

Supplementary material 1. Multiomics profiling in the observational substudy

Radiomics analysis of recurrence risk

CT imaging analysis will include preoperative and first postoperative follow-up scans preprocessed through lung segmentation, voxel standardization, resampling, and intensity normalization. We will extract features based on a 3D residual convolutional neural network (3D ResNet) and optimize them by normalized temperature-scaled cross-entropy loss. By utilizing trainable weights, we will calculate the weighted sum of 3D ResNet outputs for each view to generate the deep learning score (DLS), a predicted recurrence risk score.

Nontargeted plasma metabolomic assay

Lipids will be extracted from plasma via liquid–liquid extraction using a chloroform/methanol (2:1, v/v) solvent system. Untargeted lipidomic profiling will be performed by liquid chromatography–mass spectrometry (LC–MS) in data-dependent acquisition mode for chromatographic separation and spectral acquisition. Pooled plasma quality control (QC) samples will be analyzed throughout the sequence to monitor instrument stability and data reproducibility. Raw MS data will be processed using MS-DIAL software, with lipid identification achieved by matching MS/MS spectra against the LipidBlast in silico database. For targeted quantification, extracted ion chromatograms of predefined lipids will be integrated and normalized to internal standards using quantitative software.

Nontargeted plasma proteomic assay

Protein concentrations will be quantified via a bicinchoninic acid assay. The proteins will undergo disulfide bond reduction (10 mM dithiothreitol) and alkylation (20 mM iodoacetamide), followed by trichloroacetic acid (TCA)–acetone precipitation. Digested peptides will be analyzed by liquid chromatography–mass spectrometry (LC–MS) with data-dependent acquisition. The raw data will be processed using MaxQuant

software, and the peptides will be identified by matching against the human UniProt database.

Plasma DNA isolation and quality inspection

The plasma will be purified from peripheral blood by centrifugation at 1600 × g for 10 min at 4 °C. The plasma will be centrifuged again at 16,000 × g for 10 min at room temperature to remove any remaining cellular debris and stored at −80 °C until DNA extraction. The cfDNA isolation, concentration, and quality assessment methods were reported previously[1, 2]. The isolated cfDNA will be stored at −20 °C until library preparation. The genomic DNA of fresh frozen tissue will be extracted and purified according to the previous instructions.

CfDNA methylation assay

Matched tumor tissue, paired normal tissue, and blood samples will be analyzed by ultradeep targeted sequencing and bisulfite sequencing. cfDNA methylation detection will involve the implementation of a method called timMRD, which we developed in another study[3]. Lung cancer-specific methylation features were derived from publicly available tumor databases and our cohort data. Differentially methylated blocks (DMBs) at these CpG sites were identified using a region splitting algorithm, followed by the detection of comethylation patterns.

CfDNA genome characteristics assay

Three cfDNA genomic features (Fragment, Motif, and Bincount) will be determined through low-pass WGS detection to identify lung cancer patients. The detailed filtering process of different genome characteristics has been described previously[1].

Circulating tumor DNA assay

In this study, we will use individualized tumor-based ctDNA detection as previously reported[4]. Patient-specific somatic variants are identified by analysis of primary tumor and matched normal whole-exome sequencing (WES) samples. The

details of whole-exome library preparation, sequencing and data analysis have been reported previously[4]. On the basis of the region-specific WES data, we will combine this information to design a personalized MRD panel. Our monitoring approach tracks up to 150 patient-specific mutations and 21 key cancer driver mutations.

To capture even small amounts of tumor DNA in blood, we will use ultradeep sequencing (100,000x coverage) with at least 10 ng of ctDNA. Raw sequencing data will undergo quality control using fastp, followed by deduplication and alignment to generate BAM files. Variants with a variant allele frequency (VAF) >0.001 and ≥3 high-quality support reads will be retained for downstream analysis. Mutations with depth ≥500× and low background noise from regions surrounding specific tissue samples for personalized panel design will be selected as references to profile background mutation patterns. Permutation testing will be applied to evaluate the significance of ctDNA positivity, defined as a P value <0.01 or detection of Tier1/Tier2 de novo mutations. When we detect many mutations, we will focus first on dominant "clonal" mutations rather than minor "subclonal" mutations.

Supplementary material 2. Sample size calculation for the independent prospective cohort in the observational substudy

First, we aim to validate a multiomics lung cancer recurrence prediction model with an anticipated sensitivity of 90%±10% and a specificity of 90%±10%. To calculate the necessary sample size, we anchor our calculations to a fixed two-year recurrence (event) rate of 30%[5, 6]. Buderer's formula for sensitivity and specificity sample size estimation is used[7, 8]:

$$N = \frac{Z^2 \cdot p(1-p)}{d^2 \cdot Prevalence} = \frac{(1.96)^2 \cdot 0.9 \cdot 0.1}{0.1^2 \cdot 0.3} \approx 115\, patients$$

Here, p is the anticipated sensitivity or specificity (0.90), d is the half-width of the 95% confidence interval (0.1), and Prevalence is the event prevalence (0.3). To accommodate potential 10% attrition, at least 127 patients must be enrolled. This calculation provides the first threshold for sample size.

Second, we aim to compare the 2-year DFS rates of the predefined high-risk and low-risk groups using a two-sided log-rank test at α=0.05 and 80% power (1−β=0.80). On the basis of the published literature[5, 6] on early-stage NSCLC, we assume a 2-year DFS (recurrence or death) rate of 30% in the overall population. We further propose that the high-risk group will have a hazard ratio (HR) of 2.0 relative to the low-risk group. The Freedman formula is applied for survival comparisons:

$$N_{events} = \frac{\left(Z_{1-\frac{\alpha}{2}} + Z_{1-\beta}\right)^2}{(\ln HR)^2} = \frac{(1.96 + 0.84)^2}{(\ln 2)^2} \approx 43\, events$$

Assuming a 30% event rate, at least 144 patients are needed to observe the required number of DFS events. To accommodate potential 10% attrition, we will enroll at least 160 patients. This calculation provides the second threshold for sample size.

Supplementary material 3. Outcome and subgroup analysis in the prospective substudy

Analysis of the primary outcome

For the primary outcome, the proportion of patients without (suspected) recurrence at the two-year follow-up and the two-sided 95% Cl of the difference between the two groups will be estimated. The superiority of postoperative management for patients in the ctDNA-guided group will be established if the lower bound of the estimated 95% CI for the difference in the two-year DFS risk rate between the groups is greater than 0.5%.

We also intend to compare the primary outcome with the best historical control. Two-sided single-arm log-rank tests (α=0.05) will be used in the ctDNA-guided treatment group. For the EGFR-mutant treatment-naive cohort, the two-year DFS rate will be compared with that in the ADAURA study[9] (89% in the arm receiving osimertinib for stage IB–IIIA NSCLC); for the neoadjuvant immunotherapy cohort, the two-year DFS rate will be compared with that in the CheckMate 816 trial[10] (63.8% in the arm receiving nivolumab for stage IB–IIIA NSCLC). These analyses aim to assess the superiority of ctDNA-guided adaptive therapy over historical postoperative management.

Analysis of secondary outcomes

The secondary endpoints will be the total treatment cost within 24 months after surgery and the ICER. ICER = (treatment cost of Group A - treatment cost of Group B) ÷ (quality-adjusted life year QALY of Group A - Group B)[11]. In accordance with the WHO recommendation, 3 times our per capita gross domestic product (GDP) is used as the willingness to pay threshold, and our per capita GDP in 2023 will be $89,358; thus, the ICER threshold in this study will be $268,074/QALY.

In the secondary analysis, we will evaluate the sensitivity of the new noninvasive prediction method in a subset of the cohort, compare it with that of previous methods

based on ctDNA-MRD only, and determine the percentage of patients for whom a recurrence signal is detected by the novel method prior to clinical diagnosis and the average lead time. Afterwards, we plan to explore the relationship between ctDNA kinetic characteristics and the outcomes of neoadjuvant and adjuvant therapy (short-term efficacy measured by radiological and/or pathological response, as well as the long-term prognosis of patients).

Subgroup analysis

The primary endpoint and key secondary endpoints will be assessed in a limited number of predefined subgroups. The purpose of this analysis is to assess potential differences in outcomes across patient subgroups. The heterogeneity of effects between subgroups will be captured by logistic regression or Cox regression with corresponding forest plots and tests for interactions between treatment groups and subgroup grouping factors. The subgroup factors that will be analyzed include sex, age (>70 years versus ≤70 years), pathological type, pathological stage, lymph node metastasis, tumor EGFR status, pathological remission, tumor PD-L1 expression (≥50%, ≥1%, and <1%), and different postoperative therapies.

Supplementary material 4. Sample size estimation in the prospective substudy

Sample size calculation

A total of 756 participants will be collaboratively recruited across 10 clinical centers. Interim efficacy analysis will be performed when two-thirds of participants complete the 24-month follow-up, with the final analysis occurring upon full cohort completion. Using the O'Brien-Fleming alpha-spending function, the significance levels will be calibrated to 0.0121 (interim) and 0.046 (final) to preserve an overall 5% type I error.

Assuming uniform enrollment over 24 months and follow-up extending 24 months after enrollment, the study is powered to detect a 6% absolute improvement in two-year DFS (74.6% in the standard arm[12] [IMpower010 trial reference] vs. 80.6% in the experimental arm). The hazard rate for the standard arm is 0.293030 per 24-month interval; thus, the experimental arm is projected to achieve a corresponding hazard rate of 0.215672. Superiority testing for the hazard rate difference will be conducted by assuming an exponential model with a 0.5% superiority margin and 80% power ($1-\beta=0.80$). Sample size calculation was performed using PASS V15.0, and at least 688 patients are needed to observe the required number of DFS events. Accounting for 10% attrition, the minimum sample size was adjusted to 756.

Procedures to address nonadherence and missing data

The sample size calculation accounts for a 10% anticipated attrition rate. Missing data in the final analysis cohort will be addressed through stratified imputation based on protocol-defined data partitions. For primary efficacy endpoints, conservative approaches such as the last observation carried forwards will prioritize minimizing bias over optimistic assumptions. If the actual loss rate exceeds 10%, we will compare the baseline characteristics of completers and noncompleters to assess potential bias. Sensitivity analyses that compare complete-case and imputed datasets will be reported to demonstrate the impact of missingness on key study findings.

Supplementary material 5. Oversight monitoring of the REMODEL study

Data management

The collected data and omics results will be systematically archived in a secure database with multiple encrypted backups. Raw data retention will follow institutional guidelines. Access will be restricted to authorized researchers via password-protected hospital computers without internet connections, and all records will use anonymous study IDs. Published data will exclude any patient-sensitive information.

Adverse events (AEs) reporting and harm

The study involves data collection, imaging, and blood collection. The likelihood of serious AEs during participation in this protocol is low, although serious AEs directly related to study data collection or blood draw will be reported. Prior studies have reported <5% two-year recurrence rates in persistently MRD-negative cohorts[4, 13], suggesting a clinically acceptable relapse risk without adjuvant therapy. All patients will undergo standard postoperative monitoring, with ctDNA-MRD analysis serving as an adjunct to routine imaging. Upon radiographic progression detection (despite MRD negativity), we will immediately start guideline-directed therapeutic interventions after multidisciplinary assessment. In the case of any serious AEs, the investigator will be notified within 24 hours of occurrence, and the appropriate standardized treatment will be given in a timely manner.

References

1. Li Y, Jiang G, Wu W, Yang H, Jin Y, Wu M, Liu W, Yang A, Chervova O, Zhang S *et al*: **Multi-omics integrated circulating cell-free DNA genomic signatures enhanced the diagnostic performance of early-stage lung cancer and postoperative minimal residual disease**. *EBioMedicine* 2023, **91**:104553.
2. Abbosh C, Frankell AM, Harrison T, Kisistok J, Garnett A, Johnson L, Veeriah S, Moreau M, Chesh A, Chaunzwa TL *et al*: **Tracking early lung cancer metastatic dissemination in TRACERx using ctDNA**. *Nature* 2023, **616**(7957):553-562.
3. Chen K, Kang G, Zhang Z, Lizaso A, Beck S, Lyskjær I, Chervova O, Li B, Shen H, Wang C *et al*: **Individualized dynamic methylation-based analysis of cell-free DNA in postoperative monitoring of lung cancer**. *BMC Med* 2023, **21**(1):255.
4. Chen K, Yang F, Shen H, Wang C, Li X, Chervova O, Wu S, Qiu F, Peng D, Zhu X *et al*: **Individualized tumor-informed circulating tumor DNA analysis for postoperative monitoring of non-small cell lung cancer**. *Cancer Cell* 2023, **41**(10):1749-1762.e1746.
5. Yang B, Rao W, Luo H, Zhang L, Wang D: **Relapse-related molecular signature in early-stage lung adenocarcinomas based on base excision repair, stimulator of interferon genes pathway and tumor-infiltrating lymphocytes**. *Cancer Sci* 2020, **111**(10):3493-3502.
6. Kelsey CR, Marks LB, Hollis D, Hubbs JL, Ready NE, D'Amico TA, Boyd JA: **Local recurrence after surgery for early stage lung cancer: an 11-year experience with 975 patients**. *Cancer* 2009, **115**(22):5218-5227.
7. Malhotra RK, Indrayan A: **A simple nomogram for sample size for estimating sensitivity and specificity of medical tests**. *Indian J Ophthalmol* 2010, **58**(6):519-522.
8. Buderer NM: **Statistical methodology: I. Incorporating the prevalence of disease into the sample size calculation for sensitivity and specificity**. *Acad Emerg Med* 1996, **3**(9):895-900.
9. Tsuboi M, Herbst RS, John T, Kato T, Majem M, Grohé C, Wang J, Goldman JW, Lu S, Su WC *et al*: **Overall Survival with Osimertinib in Resected EGFR-Mutated NSCLC**. *N Engl J Med* 2023, **389**(2):137-147.
10. Forde PM, Spicer J, Lu S, Provencio M, Mitsudomi T, Awad MM, Felip E, Broderick SR, Brahmer JR, Swanson SJ *et al*: **Neoadjuvant Nivolumab plus Chemotherapy in Resectable Lung Cancer**. *N Engl J Med* 2022, **386**(21):1973-1985.
11. Toumazis I, Cao P, de Nijs K, Bastani M, Munshi V, Hemmati M, Ten Haaf K, Jeon J, Tammemägi M, Gazelle GS *et al*: **Risk Model-Based Lung Cancer Screening : A Cost-Effectiveness Analysis**. *Ann Intern Med* 2023, **176**(3):320-332.
12. Felip E, Altorki N, Zhou C, Csőszi T, Vynnychenko I, Goloborodko O, Luft A, Akopov A, Martinez-Marti A, Kenmotsu H *et al*: **Adjuvant atezolizumab after adjuvant chemotherapy in resected stage IB-IIIA non-small-cell lung cancer (IMpower010): a randomised, multicentre, open-label, phase 3 trial**. *Lancet* 2021, **398**(10308):1344-1357.
13. Zhang JT, Liu SY, Gao W, Liu SM, Yan HH, Ji L, Chen Y, Gong Y, Lu HL, Lin JT *et*

al: **Longitudinal Undetectable Molecular Residual Disease Defines Potentially Cured Population in Localized Non-Small Cell Lung Cancer**. *Cancer Discov* 2022, **12**(7):1690-1701.

The REMODEL study Consortium

Kezhong Chen[1,2,3], Guangxi Wang[4], Jiatao Zhang[5], Rong Yin[6], Ziming Li[7], Fang Wu[8], Zizi Zhou[9], Yaxing Shen[10], Yuan Cheng[11], Quanfu Huang[12], Yintao Li[13], Liang Yin[14], Xiaoju Zhu[15], Junnan Xu[16], Shanshan Yang[17]

[1]Research Unit of Intelligence Diagnosis and Treatment in Early Non-small Cell Lung Cancer, Chinese Academy of Medical Sciences, 2021RU002, Peking University People's Hospital, Beijing, 100044, China. [2]Thoracic Oncology Institute, Peking University People's Hospital, Beijing, 100044, China. [3]Department of Thoracic Surgery, Peking University People's Hospital, Beijing, 100044, China. [4]Department of Pathology, School of Basic Medical Sciences, Institute of Systems Biomedicine, Peking-Tsinghua Center for Life Sciences, Peking University Health Science Center, Beijing, P. R. China. [5]Guangdong Lung Cancer Institute, Guangdong Provincial People's Hospital, Guangdong Academy of Medical Sciences, Guangzhou, Guangdong, China. [6]Department of Thoracic Surgery, Jiangsu Cancer Hospital, Jiangsu Institute of Cancer Research, the Affiliated Cancer Hospital of Nanjing Medical University, Jiangsu Key Laboratory of Molecular and Translational Cancer Research, Collaborative Innovation Center for Cancer Personalized Medicine, Nanjing, Jiangsu, 210009, P. R. China. [7]Department of Shanghai Lung Cancer Center, Shanghai Chest Hospital, Shanghai Jiao Tong University School of Medicine, Shanghai 200030, China. [8]Department of Oncology, The Second Xiangya Hospital, Central South University, Changsha, China. [9]Department of Thoracic Surgery, National Clinical Research Center for Infectious Disease, Shenzhen Third People's Hospital, Shenzhen, China. [10]Department of Thoracic Surgery, Zhongshan Hospital, Fudan University, Shanghai, 20032, China. [11]Department of Respiratory Medicine, Peking University First Hospital, Beijing, China. [12]Department of Thoracic Surgery, Union Hospital, Tongji Medical College, Huazhong University of Science and Technology, Wuhan, China. [13]Department of Respiratory Oncology, Shandong Cancer Hospital and Institute, Shandong First Medical University and Shandong Academy of Medical Sciences, Jinan, Shandong, 250000, China. [14]School of Information and Communication Engineering, Beijing University of Posts and Telecommunications, Beijing, China. [15]Research Center for Occupational Safety and Health, National Health Commission of the People's Republic of China, Beijing, China. [16]Kanghui Biotech Co., Ltd., Liaoning, China. [17]Nanjing Geneseeq Technology Inc., Nanjing, China.
